# Diagnostic Accuracy of Presepsin and Its Impact on Early Antibiotic De-Escalation in Burn-Related Sepsis

**DOI:** 10.3390/antibiotics14080822

**Published:** 2025-08-11

**Authors:** Seontai Park, Dohern Kym, Jaechul Yoon, Yong Suk Cho, Jun Hur

**Affiliations:** 1 Department of Surgery and Critical Care, Burn Center, Hangang Sacred Heart Hospital, Hallym University Medical Center, 12, Beodeunaru-ro 7-gil, Yeongdeungpo-gu, Seoul 07247, Republic of Korea; stp89518@gamil.com (S.P.); justinoj@hallym.or.kr (J.Y.); maurchigs@hallym.or.kr (Y.S.C.); hammerj@hallym.or.kr (J.H.); 2 Burn Institutes, Hangang Sacred Heart Hospital, Hallym University Medical Center, 12, Beodeunaru-ro 7-gil, Yeongdeungpo-gu, Seoul 07247, Republic of Korea

**Keywords:** presepsin, severe burn, sepsis diagnosis, antibiotic de-escalation, antimicrobial stewardship

## Abstract

Background/Objectives: Despite overlapping inflammatory responses and frequent culture-negative results in severe burn patients, early and accurate sepsis diagnosis remains challenging. We aimed to evaluate the diagnostic performance of seven candidate biomarkers and their clinical utility, particularly in culture-negative cases. Methods: We conducted a prospective diagnostic accuracy study (January 2021–December 2022; N = 221) in the burn intensive care unit, applying a two-step feature selection to 41 candidate variables. Seven top biomarkers—presepsin, procalcitonin (PCT), albumin, C-reactive protein (CRP), prothrombin time (PT), hematocrit (Hct), and D-dimer—were measured at the moment of clinical sepsis suspicion, concurrently with blood cultures and prior to empirical antibiotic administration, within ±2 h of Sequential Organ Failure Assessment (SOFA). Diagnostic performance was evaluated using a Receiver Operating Characteristic (ROC) curve analysis to determine the area under the curve (AUC), Youden index-derived cut-offs, decision curve analysis, and Net Reclassification Improvement (NRI). Results: Presepsin achieved the highest overall AUC (0.810; 95% CI, 0.742–0.878) and outperformed other markers in culture-negative cases (AUC, 0.846 vs. 0.604; *p* = 0.015). In the decision curve analysis, presepsin and PCT maintained the largest net benefits at high thresholds, although PT, D-dimer, and Hct also retained smaller positive benefits. Patients were stratified into high- vs. low-risk groups for survival analysis using Youden index cut-offs; Cox regression confirmed PCT (Hazard Ratio 3.78; *p* < 0.001) and PT (HR 2.12; *p* = 0.018) as a significant mortality predictor, with presepsin showing borderline significance (HR 3.14; *p* = 0.055). Conclusions: The high rate of culture-negative sepsis reflects early antibiotic use suppressing culture yield rather than resistance patterns alone. Presepsin’s rapid rise and preserved accuracy under pre-sampling antibiotics suggest its value for early sepsis detection and antimicrobial stewardship. Future work will incorporate polymicrobial and multidrug-resistant bloodstream infection profiles to refine biomarker utility.

## 1. Introduction

Sepsis remains a leading cause of mortality in critically ill patients, particularly affecting those with severe burns. In burn patients with total body surface area (TBSA) involvement exceeding 20%, the risk of sepsis increases significantly due to extensive skin barrier damage and compromised immune function [1]. The Sepsis-3 criteria define sepsis as life-threatening organ dysfunction caused by a dysregulated host response to infection, quantified using the Sequential Organ Failure Assessment (SOFA) score [2]. Early diagnosis and intervention are crucial for improving outcomes in burn patients with sepsis. However, traditional diagnostic methods face significant limitations. Blood culture testing, the current gold standard, often yields delayed results and shows low sensitivity [3,4]. Moreover, early empirical antibiotic administration in burn care often leads to culture- negative results and fosters prolonged broad-spectrum use, contributing to antimicrobial resistance and underscoring the need for antimicrobial stewardship [5]. This diagnostic challenge is compounded by the overlap between infection-related symptoms and the systemic inflammatory response to burn injury itself [6].

Conventional biomarkers such as procalcitonin (PCT) and C-reactive protein (CRP) have shown limited utility in burn patients. Both markers can be elevated due to the inflammatory response to burn injury alone, leading to reduced specificity for diagnosing sepsis [7]. Although PCT has been integrated into antibiotic stewardship programs, its high false-positive rate in burn-related sepsis limits its ability to guide safe antibiotic de-escalation [8]. Similarly, CRP’s specificity is compromised by burn-induced inflammation [7].

Presepsin, also known as soluble CD14-subtype, is a 13-kDa fragment shed from the CD14 receptor on activated monocytes and macrophages, and its elevation reflects early immune response to pathogens. Recent research has identified presepsin as a promising biomarker for sepsis detection, reflecting immune cell activation in response to both pathogen- and damage-associated molecular patterns [9]. Systematic reviews and meta-analyses and confirmed presepsin’s high diagnostic accuracy across diverse sepsis cohorts [10], and its levels may indicate the appropriateness of antibiotic therapy [11]. Importantly, presepsin originates from CD14 and thus directly reflects innate immune activation in sepsis.

In addition to immune activation markers, several routinely available indices capture distinct axes of burn sepsis: prothrombin time (PT) as a surrogate of sepsis-induced coagulopathy [12]; hematocrit (Hct) to contextualize resuscitation and hematologic dynamics, with early Hct trajectories being informative in burns [13]; and D-dimer as a readout of fibrin formation and lysis that is associated with adverse outcomes in sepsis and reflects coagulation pathway activation pertinent to critical care for burns [14]. Considering PT, Hct, and D-dimer alongside presepsin/PCT/CRP may improve discrimination of infection from non-infectious inflammation and support timely decision-making in burn ICUs [12,13,14].

However, there is limited research evaluating the diagnostic accuracy of presepsin and other biomarkers specifically in severe burn patients, particularly in cases with negative blood cultures due to prior antibiotic use. Data on the utility of presepsin for guiding antibiotic de-escalation in severe burn patients are lacking despite evidence in non-burn cohorts showing that presepsin-guided protocols safely reduce antibiotic duration by 2–3 days without adverse outcomes. Understanding biomarker performance in both culture- positive and culture-negative cases is essential, given that early antibiotic administration may mask infections and affect culture results.

Therefore, this study aims to comprehensively evaluate the diagnostic value of multiple biomarkers, including presepsin, PCT, and CRP, for sepsis detection in severe burn patients. We will specifically analyze their performance in both blood culture-positive and -negative cases to determine their reliability regardless of culture outcomes. Additionally, we explore presepsin’s role within antimicrobial stewardship frameworks to optimize antibiotic prescribing and reduce resistance in burn-related sepsis.

## 2. Materials and Methods

### 2.1. Study Design and Population

**Figure 1:**
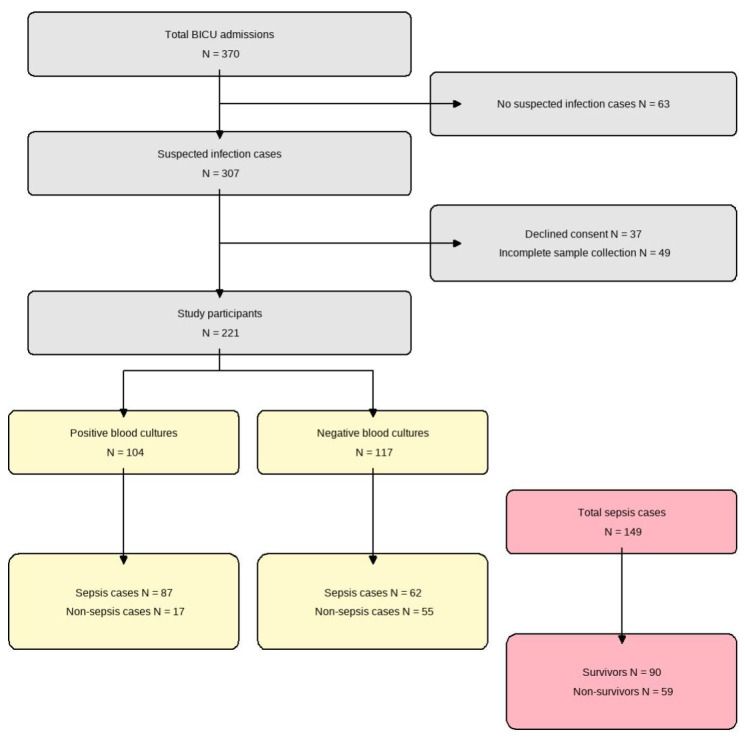
Flow diagram of patient enrollment. From 370 total admissions to the burn intensive care unit (BICU), 221 patients were included in the final analysis. These were divided into positive (*n* = 104) and negative (*n* = 117) blood culture groups. Among the total sepsis cases (*n* = 149), 90 survived and 59 did not survive.

We conducted a prospective diagnostic accuracy study at the Burn Intensive Care Unit (BICU) of Hallym University Hangang Sacred Heart Hospital between January 2021 and December 2022. Adult patients (age ≥ 18 years) with severe burns (total body surface area ≥ 20%) who developed clinically suspected sepsis during their BICU stay were eligible for inclusion. Suspected sepsis was defined as the presence of ≥2 Systemic Inflammatory Response Syndrome (SIRS) criteria in combination with clinical signs of infection. We excluded patients who (1) never developed suspected infection during admission, (2) declined to provide informed consent, or (3) had incomplete biomarker sampling or missing key laboratory data. Using Hajian-Tilaki’s method for diagnostic studies [15], we targeted a minimum sample size of 138 to achieve 90% sensitivity with ±5% precision at 95% confidence and enrolled 370 patients to allow for 20% attrition. After exclusions (63 without suspected infection, 12 patients who declined consent, and 74 patients with incomplete sampling), 221 patients remained for final analysis (Figure 1).

### 2.2. Clinical Management and Specimen Collection

On BICU admission, all patients received standard prophylactic antibiotics (third-generation cephalosporin ± aminoglycoside). Sepsis suspicion was defined by the onset of new clinical signs plus a ≥2-point rise in SOFA score. At the moment of first suspicion—but before any new or escalated empirical antibiotic dose—we drew two sets of blood cultures (10–20 mL each, aerobic + anaerobic) and simultaneously collected samples for presepsin, PCT, CRP, and albumin within a ±2-h window of SOFA assessment. All assays were performed in our central laboratory under routine quality controls, and laboratory staff remained blinded to patients’ clinical and SOFA data. No specialized research-only chemicals, devices, instruments, or commercial cell lines/samples/materials were used; all assays were performed on routine clinical analyzers in our central laboratory. Empirical broad-spectrum therapy (carbapenem + glycopeptide) was then initiated and subsequently de-escalated based on culture results.

### 2.3. Reference Standard

We defined sepsis according to the Sepsis-3 criteria [2] as our reference standard, requiring a ≥2-point increase in the Sequential Organ Failure Assessment (SOFA) score from baseline. Critical care specialists, blinded to biomarker results, performed SOFA score assessments to minimize bias. Infection was suspected when patients met 2 SIRS criteria. 

### 2.4. Biomarker Selection and Measurement

We screened 41 candidate predictors using a two-step process: (1) we conducted a univariate Receiver Operating Characteristic (ROC) analysis to calculate the AUC for each variable and (2) Random Forest modeling to rank features by mean decrease in accuracy upon permutation. Seven biomarkers consistently ranked in the top 10—presepsin, procalcitonin, albumin, C-reactive protein, prothrombin time, hematocrit, and D-dimer—were selected for final analysis (Table S1). All biomarker samples were drawn at the time of clinical sepsis suspicion, concurrent with blood cultures and before empirical therapy, within the same ±2-h window. Each analyte was measured according to standardized hospital protocols by personnel blinded to clinical and SOFA information.

### 2.5. Statistical Analysis

All statistical analyses were performed in R (version 4.3.0). Continuous variables were summarized as mean ± SD or median [IQR], depending on distribution (Shapiro–Wilk test), and compared using Student’s *t*-test or the Wilcoxon rank-sum test. Categorical variables were expressed as *n* (%) and compared using the chi-square (χ2) test or Fisher’s exact test. For diagnostic performance, we constructed ROC curves for each biomarker and calculated the area under the curve (AUC) with 95% confidence intervals. Optimal cut-off values were determined using the Youden index (sensitivity + specificity − 1). Pairwise comparisons of AUCs were conducted using DeLong’s test. To evaluate clinical utility, we performed decision curve analysis: net benefit was calculated across a range of threshold probabilities following established methods [16] and plotted separately for blood culture-positive and -negative groups. Incremental value of biomarker combinations was quantified via the Net Reclassification Improvement (NRI) and Integrated Discrimination Improvement (IDI) indices, as described by Pencina et al. [17]. For mortality prediction among sepsis patients, we fitted Cox proportional hazards models, adjusting for age, total burn surface area (TBSA), and inhalation injury. Patients were dichotomized into high- versus low-risk groups for each biomarker using the optimal Youden index cut-offs. Kaplan–Meier survival curves were then plotted for these groups and compared using the log-rank test. A two-sided *p* < 0.05 was considered statistically significant.

## 3. Results

### 3.1. Baseline Characteristics

A total of 221 patients with severe burns were included, with a median age of 56 years, and 81.0% were male. Sepsis was diagnosed in 67.4% (149/221) of patients, and the overall mortality rate was 33.0% (73/221). Patients were categorized into blood culture-positive (47.1%, 104/221) and culture-negative groups (52.9%, 117/221). Compared to the culture-negative group, the culture-positive patients had a higher incidence of sepsis (83.7% vs. 53.0%, *p* < 0.001), higher mortality rate (49.0% vs. 18.8%, *p* < 0.001), larger median total body surface area (TBSA) burned (44% vs. 25%, *p* < 0.001), and higher severity scores (Table 1).

### 3.2. Distribution of Bacterial Species and Antibiotic Resistance

**Figure 2:**
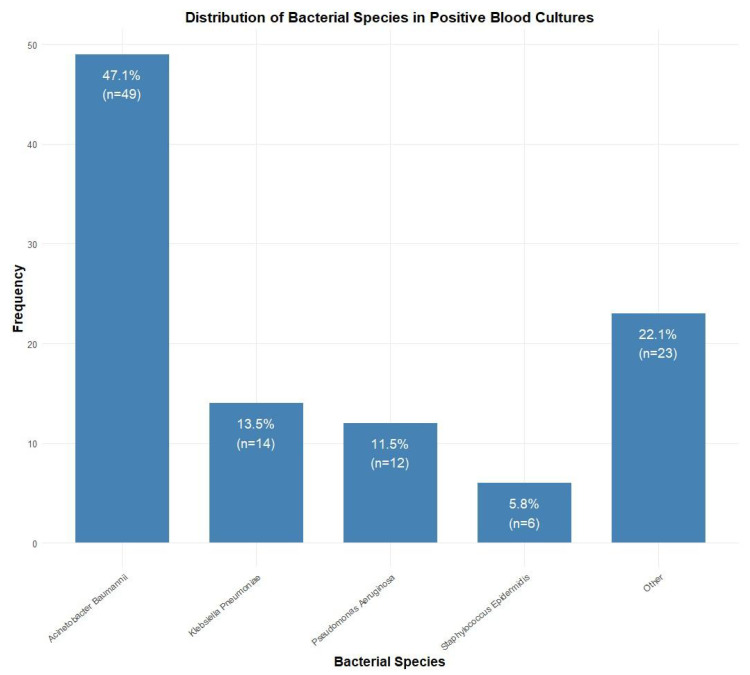
Distribution of bacterial species isolated from positive blood cultures (*n* = 104). *Acinetobacter baumannii* was the most common isolate (47.1%, *n* = 49), followed by *Klebsiella pneumoniae* (13.5%, *n* = 14), *Pseudomonas aeruginosa* (11.5%, *n* = 12), and *Staphylococcus epidermidis* (5.8%, *n* = 6).

**Figure 3:**
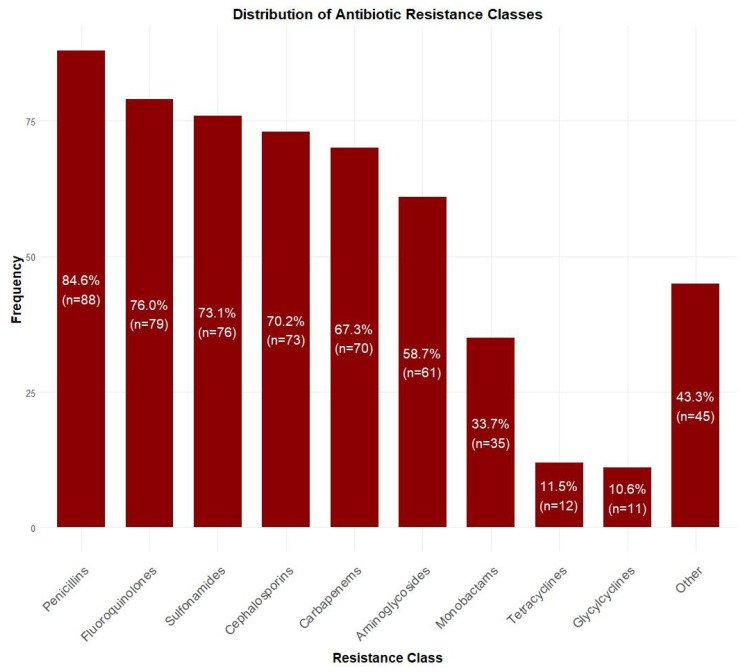
Distribution of antibiotic resistance patterns among isolated bacteria. Highest resistance was observed for penicillins (84.6%, *n* = 88), followed by fluoroquinolones (76.0%, *n* = 79) and sulfonamides (73.1%, *n* = 76), while lowest resistance was seen for glycylcyclines (10.6%, *n* = 11).

Among blood culture-positive specimens (*n* = 104), Acinetobacter baumannii was the most prevalent pathogen (47.1%, *n* = 49), followed by Klebsiella pneumoniae (13.5%, *n* = 14), Pseudomonas aeruginosa (11.5%, *n* = 12), and Staphylococcus epidermidis (5.8%, *n* = 6). Other species accounted for 22.1% (*n* = 23) of isolates (Figure 2). Antibiotic resistance rates were the highest for penicillins (84.6%, *n* = 88), followed by fluoroquinolones (76.0%, *n* = 79), sulfonamides (73.1%, *n* = 76), cephalosporins (70.2%, *n* = 73), and carbapenems (67.3%, *n* = 70). Lower resistance rates were observed for aminoglycosides (58.7%, *n* = 61), monobactams (33.7%, *n* = 35), tetracyclines (11.5%, *n* = 12), and glycylcyclines (10.6%, *n* = 11) (Figure 3).

### 3.3. Diagnostic Performance of Biomarkers

**Figure 4:**
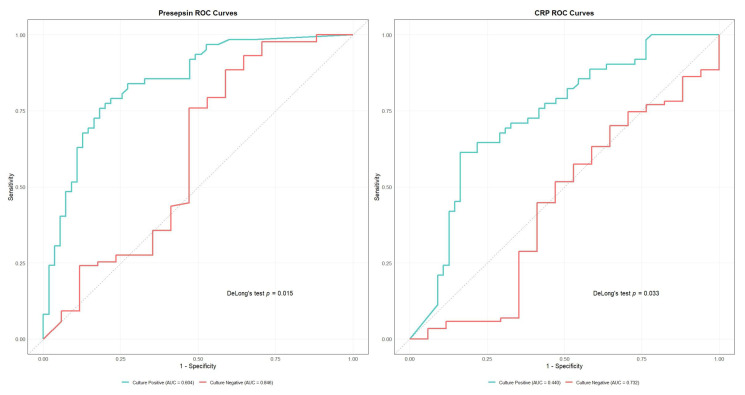
Receiver Operating Characteristic (ROC) curves comparing diagnostic performance between culture-positive and culture-negative cases for presepsin (**left**) and C-reactive protein (CRP) (**right**). Presepsin showed superior performance in culture-negative cases (area under the curve [AUC] = 0.846 vs. 0.604, *p* = 0.015). CRP also demonstrated better performance in culture-negative cases (AUC = 0.732 vs. 0.440, *p* = 0.003).

Presepsin demonstrated the highest overall diagnostic accuracy (AUC 0.810, 95% CI: 0.742–0.878), with optimal sensitivity of 82.6% and specificity of 72.2% at a cutoff value of 472.0 pg/mL (Table 2). Other biomarkers showed moderate diagnostic performance, namely procalcitonin (AUC 0.752), albumin (AUC 0.750), and CRP (AUC 0.692). PT (AUC 0.685), hematocrit (AUC 0.669), and D-dimer (AUC 0.676) demonstrated lower but significant diagnostic value (Figure S1, Table 2). DeLong’s test confirmed presepsin’s superior performance against all biomarkers except procalcitonin and albumin (Table 3). For subgroup analysis, we plotted ROC curves only for presepsin and CRP—the only two biomarkers whose AUC differences between culture-negative and culture-positive groups reached statistical significance according to DeLong’s test (presepsin, *p* = 0.015; CRP, *p* = 0.003)—the results are shown in Figure 4 and Table S2. Subgroup AUCs for the remaining markers are provided in Table 2.

### 3.4. Decision Curve Analysis

**Figure 5:**
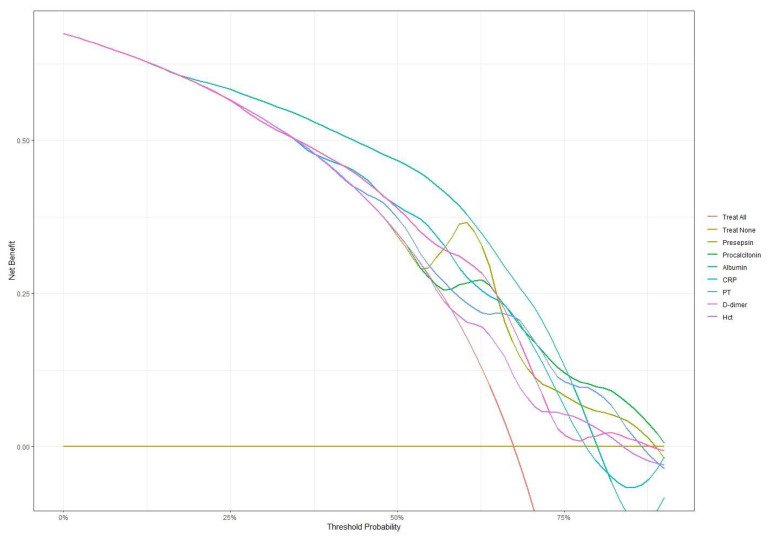
Decision curve analysis comparing net benefit across different threshold probabilities for all biomarkers. Presepsin and procalcitonin maintained higher net benefits at higher threshold probabilities compared to other markers.

Decision curve analysis was performed to assess the net benefit of each biomarker across threshold probabilities in the overall cohort, as well as in the culture-negative and culture-positive subgroups. In the overall cohort (Figure 5, Table S3), all biomarkers achieved similar net benefits (0.535–0.559) at low thresholds (0.20–0.30). As the thresholds increased to 0.40–0.60, presepsin led with a net benefit of 0.412 at 0.60, followed by albumin (0.385) and procalcitonin (0.299). Even at high thresholds (0.75–0.80), presepsin maintained net benefits of 0.090–0.050, and those for procalcitonin were 0.113–0.100; PT, D-dimer, and hematocrit also retained small positive net benefits (0.02–0.05). In the culture-negative subgroup (Figure S2; Table S4), presepsin again demonstrated the highest net benefits at low thresholds (0.412–0.373) and peaked at an intermediate threshold of 0.40 (0.348), uniquely preserving a positive net benefit of 0.077 at high thresholds (0.75–0.80). Procalcitonin remained stable at high thresholds (0.137–0.120), whereas albumin, CRP, PT, D-dimer, and hematocrit fell below zero at high thresholds, underscoring presepsin’s distinct utility when cultures are negative. Conversely, in the culture-positive subgroup (Figure S3; Table S5), all biomarkers delivered high net benefits at low thresholds (0.796–0.703) and sustained positive benefits above 0.317 up to a threshold of 0.75. At a high threshold (0.85), presepsin (0.093), procalcitonin (0.122), hematiocrit (0.0151), and albumin (0.106) led the set, while PT and D-dimer exhibited smaller positive net benefits.

### 3.5. Reclassification Analysis

Albumin showed significant improvements in both IDI (0.142, *p* < 0.001) and NRI (0.609, *p* < 0.001) compared to presepsin alone. Procalcitonin maintained comparable discriminative improvement (IDI −0.005, *p* = 0.737) but showed lower reclassification capability (NRI −0.615, *p* < 0.001). D-dimer demonstrated significantly lower discriminative ability (IDI −0.048, *p* < 0.01; NRI −0.662, *p* < 0.001) (Table 4). In the blood culture-negative group, albumin showed significant improvement in NRI (0.434, *p* = 0.016) compared to presepsin, while D-dimer demonstrated significantly lower IDI (−0.136, *p* < 0.001) and NRI (−0.838, *p* < 0.001) (Table S6). In the blood culture-positive group, albumin showed significant improvement in IDI (0.068, *p* < 0.05), while PT demonstrated significant improvement in NRI (0.679, *p* < 0.01). Other biomarkers showed no significant differences in either IDI or NRI (Table S7).

### 3.6. Mortality Prediction

Among sepsis patients (*n* = 149), the overall mortality was 39.6% (59 deaths; Table S8). In Cox regression adjusted for age, total burn surface area, and inhalation injury, elevated procalcitonin (HR 3.78; 95% CI, 1.89–7.55; *p* < 0.001) and prolonged prothrombin time (HR 2.12; 95% CI, 1.14–3.96; *p* = 0.018) were independently associated with higher mortality, while presepsin showed borderline significance (HR 3.14; 95% CI, 0.97–10.14; *p* = 0.055) (Table 5). Kaplan–Meier curves stratified by each biomarker’s Youden index cut-off—using the same adjustment factors—confirmed significantly poorer survival in the high-risk procalcitonin and PT groups (Figure S4).

## 4. Discussion

This prospective study provides comprehensive evidence for the diagnostic utility of multiple biomarkers in detecting sepsis among severe burn patients. Our findings demonstrate four key insights: the superior diagnostic performance of presepsin, particularly in blood culture-negative cases; distinct utility patterns of biomarkers across different clinical thresholds; the complementary value of combining specific biomarkers; and the impact of high antimicrobial resistance on diagnostic strategies.

Presepsin emerged as the leading diagnostic biomarker in our cohort (AUC 0.810; 95% CI, 0.742–0.878), with significantly higher performance in blood culture-negative cases (AUC 0.846 vs. 0.604; *p* = 0.015). This phenomenon is driven by presepsin’s rapid kinetics—levels begin to rise within 1 h of pathogen exposure and peak by approximately 3 h, and the plasma half-life is 4–5 h [18]. In burn patients, early empirical antibiotics can reduce blood culture positivity by more than 50% within 2 h of administration; however, they do not blunt the rise of presepsin, preserving its diagnostic utility in culture-negative sepsis [19]. Mechanistically, presepsin (sCD14-ST) is generated via cathepsin D-mediated cleavage of CD14 on activated monocytes/macrophages and also via phagocytosis-independent CD14 cleavage pathways in sterile inflammation settings [11,20]. As infections become established and cultures turn positive, regulatory feedback may attenuate CD14 shedding and thus reduce presepsin levels, diminishing its discrimination in culture-positive cases. These combined features explain why presepsin yields superior AUCs in culture-negative sepsis yet is less discriminative when cultures are positive. The higher recall rate in culture-negative cases (0.892 vs. 0.837) further supports presepsin’s utility in this challenging diagnostic scenario. The integration of presepsin measurement into antibiotic stewardship protocols could enable earlier, evidence-based adjustment of empirical therapy—thereby supporting tailored antimicrobial therapy and duration in line with IDSA and SHEA stewardship recommendations [21]. The decision curve analysis revealed crucial patterns in clinical utility across different thresholds. In culture-negative cases, presepsin demonstrated consistently superior net benefit (0.412–0.373) at low thresholds (0.20–0.25) and maintained a positive net benefit (0.077) at high thresholds (0.75–0.80), as did procalcitonin (net benefit 0.120–0.100). This sustained performance across thresholds suggests that both presepsin and PCT can reliably support screening and confirmation in culture-negative sepsis. In culture-positive cases, while multiple biomarkers showed similar performance at lower thresholds (net benefits 0.796–0.703), only presepsin (0.093), procalcitonin (0.122), and albumin (0.106) maintained meaningful utility at high thresholds (>0.85), indicating their specific value in severe cases. Clinically, a threshold probability represents the predicted risk above which clinicians continue broad-spectrum antibiotics. At a 0.4 threshold (40% predicted sepsis risk), decision curve analysis indicates that de-escalation—narrowing spectrum or discontinuation—provides greater net benefit than “treat-all,” as the harm of unnecessary antibiotics outweighs the risk of missed infections [22]. At a higher threshold of 0.6, clinicians require greater certainty; a predicted risk below 0.6 supports intermediate strategies (e.g., switch to narrower agents or shorter courses) with close monitoring, balancing efficacy and stewardship goals [21,22]. These thresholds parallel procalcitonin-guided protocols, which use similar cut-offs (0.25–0.5 ng/mL) to guide antibiotic discontinuation or narrowing in sepsis management [8]. Finally, combining presepsin and PCT measurements may further refine risk stratification and enable a “double-trigger” approach—requiring both a presepsin level below 472 pg/mL and clinical stability—to safely guide de-escalation at 48–72 h post-suspected sepsis.

The complementary roles of biomarkers were further elucidated through reclassification analyses. Albumin significantly improved both discriminative ability (IDI 0.142, *p* < 0.001) and risk reclassification (NRI 0.609, *p* < 0.001) compared to presepsin alone. This synergistic effect showed context-specific patterns, with albumin demonstrating significant NRI improvement (0.434, *p* = 0.016) in culture-negative cases and IDI improvement (0.068, *p* < 0.05) in culture-positive cases. These findings suggest that optimal biomarker combinations may differ based on blood culture status. These decision curve profiles suggest clinically actionable thresholds for presepsin that align with stewardship objectives, balancing early antibiotic initiation against de-escalation opportunities to minimize unnecessary broad-spectrum exposure [22].

Our microbiological findings highlight the considerable challenges posed by multidrug-resistant infections in the burn unit. Acinetobacter baumannii predominated (47.1%), with very high resistance rates to penicillins (84.6%), fluoroquinolones (76.0%), and sulfonamides (73.1%). However, the high proportion of culture-negative sepsis in our cohort is explained primarily by the early administration of broad-spectrum antibiotics—often before blood cultures are obtained—which reduces culture positivity by over 50% [19]. In this context, presepsin remains elevated despite pre-sampling antibiotic exposure, thereby preserving its diagnostic accuracy in culture-negative cases. Consequently, stewardship programs should tailor empirical regimens to local antibiograms while leveraging presepsin measurement to minimize broad-spectrum antibiotic use and curb further resistance emergence [23].

Individual biomarkers demonstrated distinct prognostic capabilities. Procalcitonin showed the strongest association with mortality (HR 3.781, *p* < 0.001), aligning with previous findings linking elevated levels to poor outcomes in sepsis [8]. While its diagnostic performance was lower than presepsin, particularly in culture-negative cases, procalcitonin’s consistent performance across thresholds suggests its value in severity assessment. PT’s significant mortality association (HR 2.123, *p* = 0.018) reflects the prognostic importance of coagulation dysfunction in sepsis [12], while presepsin’s borderline significance in mortality prediction (HR 3.143, *p* = 0.055) suggests that its primary utility lies in early diagnosis rather than prognostication.

Conventional markers showed varying limitations. CRP demonstrated lower diagnostic performance compared to presepsin and procalcitonin, likely due to its non-specific elevation in burn-related inflammation [24]. Albumin, while valuable in risk stratification, can be confounded by nutritional status, fluid resuscitation, and liver function [25,26]. D-dimer and hematocrit showed limited prognostic value, likely due to their susceptibility to various confounding factors in burn patients [13,14].

Although our current analysis did not stratify culture-positive isolates by polymicrobial involvement or multidrug-resistant profiles due to resource constraints, polymicrobial bloodstream infections and MDR pathogens are common in burn patients and associated with worse outcomes. Such complex infections further complicate empirical antibiotic selection and may alter biomarker kinetics. We plan to perform detailed characterization of polymicrobial and MDR patterns in future studies to fully elucidate presepsin’s diagnostic and prognostic utility in these challenging scenarios.

Our study has several important limitations. First, as a single-center investigation in a specialized burn unit population, our findings may not be generalizable to other settings or patient groups. Second, we did not perform serial biomarker measurements, which prevents an analysis of how marker levels evolve over time in response to therapy. Third, although we identified threshold-dependent diagnostic performance using the Youden index, these internally derived cut-offs (e.g., presepsin 472 pg/mL) lack external validation, and multicenter cohorts are needed to confirm their applicability. Fourth, all patients received prophylactic antibiotics on admission; while we ensured that study-related sampling occurred before any new empirical dosing, pre-treatment effects may still have suppressed culture yield and altered biomarker kinetics—future work should thus include sensitivity analyses stratified by recent antibiotic exposure. Fifth, we did not include a true non-sepsis control group of burn patients without suspected infection (no presepsin data remain for the 63 excluded non-sepsis cases), making it impossible to distinguish baseline post-burn inflammation from sepsis-related elevations. Finally, we did not assess the downstream clinical impact of presepsin-guided stewardship—such as reductions in antibiotic days of therapy or spectrum narrowing—which must be evaluated in dedicated interventional trials.

## 5. Conclusions

This comprehensive evaluation demonstrates presepsin’s superior diagnostic value for sepsis in severe burn patients, particularly in challenging culture-negative cases. Its performance characteristics vary meaningfully across different clinical thresholds and blood culture status, suggesting the need for context-specific implementation. The complementary roles of different biomarkers, especially the synergistic effect of presepsin with albumin, support a combined approach to sepsis diagnosis. In the context of high antimicrobial resistance and the limitations of traditional diagnostics, presepsin-based strategies could improve both diagnostic accuracy and antibiotic stewardship. Future prospective, multi- center ASP trials are warranted to validate presepsin-based thresholds, quantify reductions in antibiotic duration and spectrum, and measure downstream effects on resistance rates and patient outcomes in burn-related sepsis. The integration of these findings into clinical protocols could ultimately lead to more precise and timely interventions in burn-related sepsis management.

## Figures and Tables

**Table 1:** Baseline characteristics of all patients by blood culture status: implications for sepsis diagnosis.

Group Variables		Overall, *n* = 221	Negative Blood Culture, *n* = 117 (52.9%)	Positive Blood Culture, *n* = 104 (47.1%)	*p*-Value
Outcomes	Mortality	73 (33.0%)	22 (18.8%)	51 (49.0%)	<0.001
	Sepsis 3	149 (67.4%)	62 (53.0%)	87 (83.7%)	<0.001
	Age	56 [43, 66]	57 [45, 68]	56 [38, 64]	0.341
	Sex				0.732
	Male	179 (81.0%)	96 (82.1%)	83 (79.8%)	
Baseline	Female	42 (19.0%)	21 (17.9%)	21 (20.2%)	
	TBSA (%)	38 [20, 55]	25 [15, 43]	44 [33, 63]	<0.001
	Inhalation				0.081
	No	181 (81.9%)	101 (86.3%)	80 (76.9%)	
	Yes	40 (18.1%)	16 (13.7%)	24 (23.1%)	
	Type				0.306
	FB	162 (73.3%)	85 (72.6%)	77 (74.0%)	
Baseline (cont.)	SB	25 (11.3%)	10 (8.5%)	15 (14.4%)	
	EB	21 (9.5%)	14 (12.0%)	7 (6.7%)	
	ChB	2 (0.9%)	2 (1.7%)	0 (0.0%)	
	CoB	11 (5.0%)	6 (5.1%)	5 (4.8%)	
	LOICU (days)	21 [12, 33]	18 [8, 28]	24 [17, 40]	<0.001
	Cardiovascular disease	36 (16.3%)	19 (16.2%)	17 (16.3%)	>0.999
	Endocrine disorders	21 (9.5%)	13 (11.1%)	8 (7.7%)	0.492
Medical history	Malignant	10 (4.5%)	4 (3.4%)	6 (5.8%)	0.522
	Others	4 (1.8%)	3 (2.6%)	1 (1.0%)	0.624
	Operations	81 (36.7%)	44 (37.6%)	37 (35.6%)	0.781
	ABSI	9 [7, 10]	8 [7, 9]	9 [8, 11]	<0.001
	rBaux	97 [81, 112]	90 [76, 100]	106 [94, 119]	<0.001
Severity scores	Hangang	131 [120, 144]	126 [117, 136]	137 [126, 152]	<0.001
	APACHE IV	60 [34, 83]	51 [29, 76]	67 [42, 88]	0.001
	SOFA	4 [2, 7]	3 [2, 5]	6 [3, 9]	<0.001
Complications	ARDS	68 (30.8%)	27 (23.1%)	41 (39.4%)	0.013
	AKI	64 (29.0%)	25 (21.4%)	39 (37.5%)	0.011
Interventions	Ventilator	125 (56.6%)	51 (43.6%)	74 (71.2%)	<0.001
	CRRT	47 (21.3%)	13 (11.1%)	34 (32.7%)	<0.001
	Presepsin	754 [362, 1758]	467 [249, 1042]	1299 [556, 3538]	<0.001
	Procalcitonin	0.5 [0.2, 1.5]	0.4 [0.2, 0.7]	0.8 [0.3, 3.7]	<0.001
	Albumin	2.50 [2.30, 2.80]	2.60 [2.40, 3.00]	2.50 [2.20, 2.70]	<0.001
Biomarkers	CRP	117 [67, 181]	98 [60, 151]	136 [77, 191]	0.006
	PT	14.70 [13.70, 16.10]	14.30 [13.50, 15.30]	15.25 [14.08, 17.00]	<0.001
	D-dimer	2.0 [1.1, 2.8]	1.9 [1.0, 2.7]	2.3 [1.4, 3.2]	0.019
	Hct	28 [25, 33]	30 [27, 37]	27 [25, 30]	<0.001

Note: Data are presented as *n* (%) or median [interquartile range]. Abbreviations: IQR, interquartile range; TBSA, total body surface area; FB, flame burn; SB, scald burn; EB, electrical burn; ChB, chemical burn; CoB, contact burn; LOICU, length of intensive care unit stay; ABSI, Abbreviated Burn Severity Index; rBaux, revised Baux score; APACHE IV, Acute Physiology and Chronic Health Evaluation IV; SOFA, Sequential Organ Failure Assessment; ARDS, acute respiratory distress syndrome; AKI, acute kidney injury; CRRT, continuous renal replacement therapy; CRP, C-reactive protein; PT, prothrombin time; Hct, hematocrit.

**Table 2:** Diagnostic performance of predictors for Sepsis_3 in overall and subgroup analyses.

Biomarkers	Group	AUC (95% CI)	DeLong’s	Cutoff	Sensitivity	Specificity	PPV	NPV	Youden
			***p*-Value**		**(95% CI)**	**(95% CI)**	**(95% CI)**	**(95% CI)**	**Index**
	Overall	0.810		472	0.826	0.722	0.860	0.667	0.548
Presepsin		(0.742–0.878)	0.015 *		(0.980–1.000)	(0.861–1.000)	(0.790–0.910)	(0.550–0.767)	
	Culture-	0.604		458	0.885	0.412	0.885	0.412	0.297
	Positive	(0.425–0.783)			(0.782–1.000)	(0.588–1.000)	(0.794–0.941)	(0.194–0.665)	
			
	Culture-	0.846		472	0.758	0.818	0.825	0.750	0.576
	Negative	(0.775–0.917)			(0.965–1.000)	(0.927–1.000)	(0.696–0.908)	(0.619–0.849)	
	Overall	0.752		0.66	0.517	0.903	0.917	0.474	0.420
Procalcitonin		(0.686–0.819)	0.849		(0.922–1.000)	(0.917–1.000)	(0.830–0.963)	(0.389–0.561)	
	Culture-	0.701		0.26	0.839	0.588	0.912	0.417	0.427
	Positive	(0.537–0.865)			(0.862–1.000)	(0.706–1.000)	(0.823–0.961)	(0.228–0.631)	
	Culture-	0.719		0.66	0.403	0.964	0.926	0.589	0.367
	Negative	(0.628–0.810)			(0.875–1.000)	(0.964–1.000)	(0.742–0.987)	(0.480–0.690)	
	Overall	0.750		2.8	0.919	0.542	0.235	0.194	0.461
Albumin		(0.673–0.828)	0.243		(0.967–1.000)	(0.787–1.000)	(0.132–0.378)	(0.139–0.263)	
	Culture-	0.647		2.8	0.874	0.412	0.611	0.116	0.285
	Positive	(0.477–0.817)			(0.841–1.000)	(0.647–1.000)	(0.361–0.817)	(0.060–0.208)	
	Culture-	0.762		2.8	0.871	0.636	0.186	0.270	0.507
	Negative	(0.670–0.855)			(0.935–1.000)	(0.764–1.000)	(0.089–0.339)	(0.177–0.388)	
	Overall	0.692	0.003	114.8	0.617	0.722	0.821	0.477	0.340
CRP		(0.613–0.771)	**		(0.893–1.000)	(0.792–1.000)	(0.735–0.885)	(0.381–0.574)	
	Culture-	0.440		179.2	0.701	0.353	0.812	0.153	0.054
	Positive	(0.273–0.608)			(0.552–1.000)	(0.412–1.000)	(0.630–0.921)	(0.082–0.261)	
	Culture-	0.732		119.9	0.613	0.836	0.809	0.657	0.449
	Negative	(0.638–0.825)			(0.871–1.000)	(0.800–1.000)	(0.663–0.904)	(0.533–0.764)	
	Overall	0.685		14.9	0.570	0.792	0.850	0.471	0.362
PT		(0.612–0.759)	0.787		(0.814–1.000)	(0.889–1.000)	(0.761–0.911)	(0.380–0.564)	
	Culture-	0.639		14.7	0.644	0.647	0.903	0.262	0.291
	Positive	(0.493–0.784)			(0.733–1.000)	(0.706–1.000)	(0.795–0.960)	(0.144–0.423)	
	Culture-	0.663		14.9	0.516	0.818	0.762	0.600	0.334
	Negative	(0.565–0.762)			(0.774–1.000)	(0.836–1.000)	(0.602–0.874)	(0.480–0.709)	
	Overall	0.676		1.41	0.765	0.514	0.765	0.514	0.279
D-dimer		(0.601–0.752)	0.776		(0.883–1.000)	(0.847–1.000)	(0.687–0.829)	(0.394–0.632)	
	Culture-	0.645		1.04	0.851	0.412	0.881	0.350	0.262
	Positive	(0.488–0.802)			(0.770–1.000)	(0.706–1.000)	(0.788–0.938)	(0.163–0.591)	
	Culture-	0.672		1.42	0.742	0.527	0.639	0.644	0.269
	Negative	(0.574–0.770)			(0.871–1.000)	(0.836–1.000)	(0.517–0.746)	(0.487–0.777)	

	Overall	0.669		32.2	0.846	0.486	0.397	0.227	0.332
Hct		(0.590–0.748)	0.264		(0.899–1.000)	(0.842–1.000)	(0.273–0.534)	(0.167–0.300)	
	Culture-	0.565		24.9	0.287	0.941	0.795	0.038	0.229
	Positive	(0.426–0.705)			(0.586–1.000)	(0.824–1.000)	(0.685–0.875)	(0.002–0.216)	
	Culture-	0.663		32.9	0.806	0.564	0.279	0.324	0.370
	Negative	(0.562–0.765)			(0.871–1.000)	(0.727–1.000)	(0.158–0.439)	(0.223–0.444)	

Note: Significance codes: * *p* < 0.05, ** *p* < 0.01. Values are presented with 95% confidence intervals in parentheses. DeLong’s p-value compares AUCs between culture-positive and culture-negative groups. Abbreviations: AUC, area under the curve; CI, confidence interval; PPV, positive predictive value; NPV, negative predictive value.

**Table 3:** Comparison of ROC curves with presepsin (AUC = 0.810) using DeLong’s test.

Biomarker	Comparator AUC	AUC Difference	*p*-Value
Procalcitonin	0.752	−0.058	0.142
Albumin	0.750	−0.060	0.121
CRP	0.692	−0.118	0.006 **
PT	0.685	−0.125	0.005 **
D-dimer	0.676	−0.134	0.005 **
Hct	0.669	−0.141	<0.001 ***

Note: Significance codes: ** *p* < 0.01, *** *p* < 0.001. Abbreviations: AUC, area under the curve; CRP, C-reactive protein; PT, pro-thrombin time; Hct, hematocrit.

**Table 4:** Comparison of IDI and NRI with presepsin.

Biomarker	IDI (95% CI)	*p*-Value	NRI (95% CI)	*p*-Value
Procalcitonin	−0.005 (−0.242~0.232)	0.737	−0.615 (−1.319~0.089)	<0.001 ***
Albumin	0.142 (−0.195~0.479)	<0.001 ***	0.609 (−0.115~1.332)	<0.001 ***
CRP	0.016 (−0.275~0.307)	0.460	0.253 (−0.486~0.992)	0.075
PT	0.003 (−0.269~0.275)	0.868	−0.048 (−0.790~0.695)	0.738
D-dimer	−0.048 (−0.309~0.213)	0.007 **	−0.662 (−1.328~0.004)	<0.001 ***
Hct	0.021 (−0.279~0.321)	0.369	0.248 (−0.490~0.987)	0.081

Note: Significance codes: ** *p* < 0.01, *** *p* < 0.001, Abbreviations: IDI, integrated discrimination improvement; NRI, net reclassification improvement; CI, confidence interval; CRP, C-reactive protein; PT, prothrombin time; Hct, hematocrit.

**Table 5:** Cox proportional hazards analysis for mortality according to biomarker levels.

Biomarker	Hazard Ratio (95% CI)	*p*-Value
Presepsin	3.143 (0.974–10.143)	0.055
Procalcitonin	3.781 (1.893–7.552)	<0.001 ***
Albumin	3.880 (0.533–28.233)	0.181
CRP	0.804 (0.459–1.405)	0.443
PT	2.123 (1.137–3.961)	0.018 *
D-dimer	0.716 (0.400–1.282)	0.262
Hct	0.731 (0.309–1.731)	0.476

Note: Significance codes: *** *p* < 0.001, and * *p* < 0.05. Abbreviations: CI, confidence interval; CRP, C-reactive protein; PT, prothrombin time; Hct, hematocrit.

## Data Availability

The data presented in this study are available upon request from the corresponding author due to patient privacy restrictions.

## Supplementary Materials

The following supporting information can be downloaded at https://www.mdpi.com/article/10.3390/antibiotics14080822/s1. The supplementary figures (Figure S1–S4) and tables (Table S1–S8) provide additional information regarding the diagnostic performance, decision curve analysis, and survival data that were pivotal to the study.
