# Supplementary material for: Diagnostic Accuracy of Presepsin and Its Impact on Early Antibiotic De-Escalation in Burn-Related Sepsis
Source: Antibiotics (Basel). 2025 Aug 11;14(8):822. doi: 10.3390/antibiotics14080822 (PMC12383196; doi:10.3390/antibiotics14080822)
Supplement: Supplementary file 1 [file antibiotics-14-00822-s001.zip › antibiotics-3739983-supplementary.pdf]

Supplementary material

Diagnostic Accuracy of Presepsin and Its Impact on Early Antibiotic De-Escalation in Burn-Related Sepsis

Seontai Park [1], Dohern Kym [1,2,*], Jaechul Yoon [1], Yong Suk Cho [1,2] and Jun Hur [1,2]

1 Department of Surgery and Critical Care, Burn Center, Hangang Sacred Heart Hospital, Hallym University Medical Center, 12, Beodeunaru-ro 7-gil, Yeongdeungpo-gu, Seoul 07247, Republic of Korea; stp89518@gamil.com (S.P.); justinoj@hallym.or.kr (J.Y.); maurchigs@hallym.or.kr (Y.S.C.); hammerj@hallym.or.kr (J.H.)

2 Burn Institutes, Hangang Sacred Heart Hospital, Hallym University Medical Center, 12, Beodeunaru-ro 7-gil, Yeongdeungpo-gu, Seoul 07247, Republic of Korea

* Correspondence: dohern@hallym.or.kr; Tel.: +82-2-2639-5446

Contents

Figure S1. ROC Curves Comparing Diagnostic Performance of All Biomarkers for Sepsis Detection. 3

Figure S2. Decision Curve Analysis for Diagnostic Biomarkers in Sepsis Detection Among Burn Patients with Negative Blood Cultures. 4

Figure S3. Decision Curve Analysis for Diagnostic Biomarkers in Sepsis Detection Among Burn Patients with Positive Blood Cultures. 5

Figure S4. Kaplan–Meier Survival Curves Comparing High and Low Biomarker Groups Over 60 Days. 6

Table S1. Comparison of Random Forest and AUC Analysis Results for Biomarker Selection 7

Table S2. ROC Curves by Culture Status Whose AUC Differences Between Culture-Negative and Culture-Positive Groups Reached Statistical Significance. 9

Table S3. Net Benefit at Different Threshold Probabilities in Blood CulturesOverall 10

Table S4. Net Benefit at Different Threshold Probabilities in Negative Blood Culture 11

Table S5. Net Benefit at Different Threshold Probabilities in Positive Blood Culture 12

Table S6. Comparison of IDI and NRI with Presepsin in Negative Blood Culture 13

Table S7. Comparison of IDI and NRI with Presepsin in Positive Blood Culture 14

Table S8. Clinical Characteristics of Sepsis Patients: Implications for Mortality Prediction... 15

Figure S1. ROC curves comparing diagnostic performance of all biomarkers for sepsis detection.

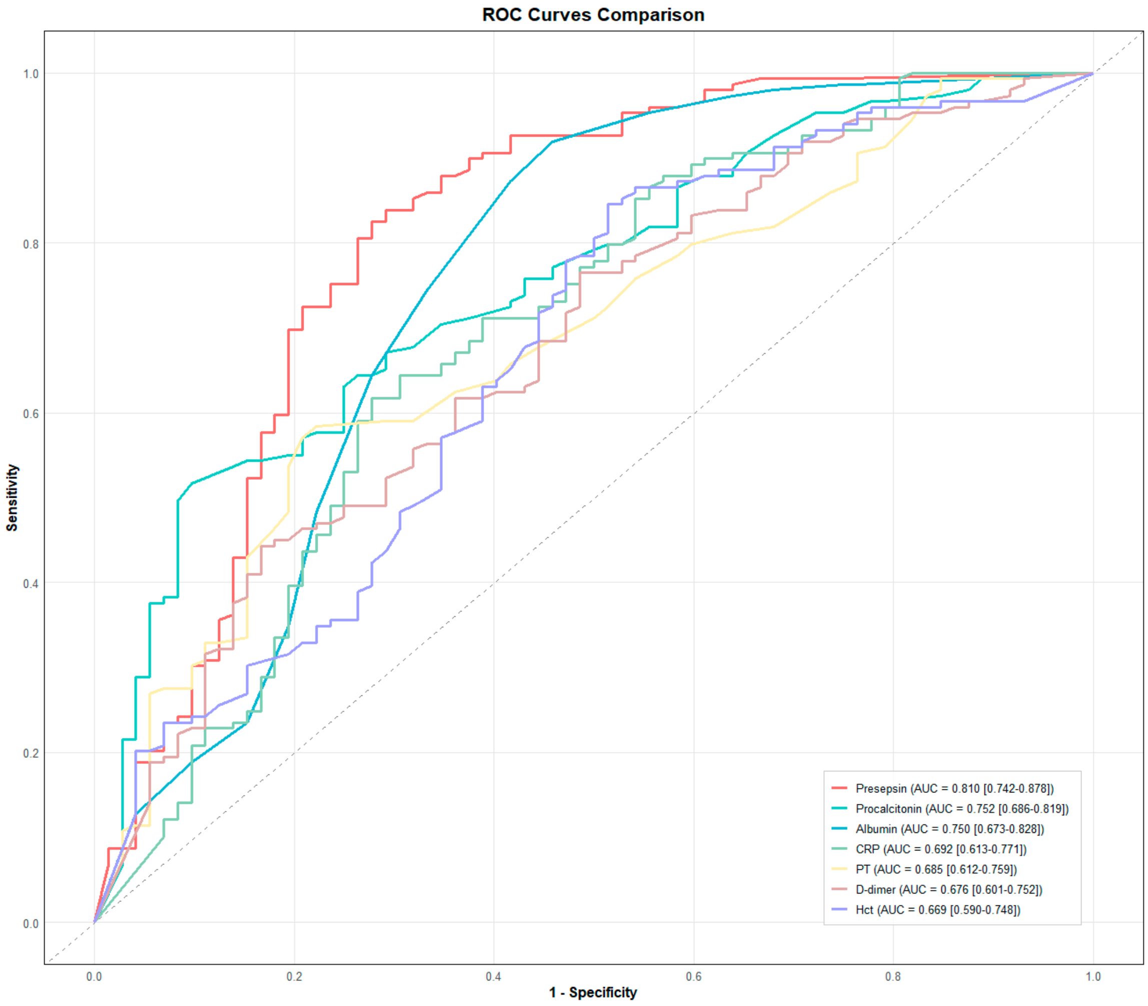

Figure S2. Decision curve analysis for diagnostic biomarkers in sepsis detection among burn patients with negative blood cultures.

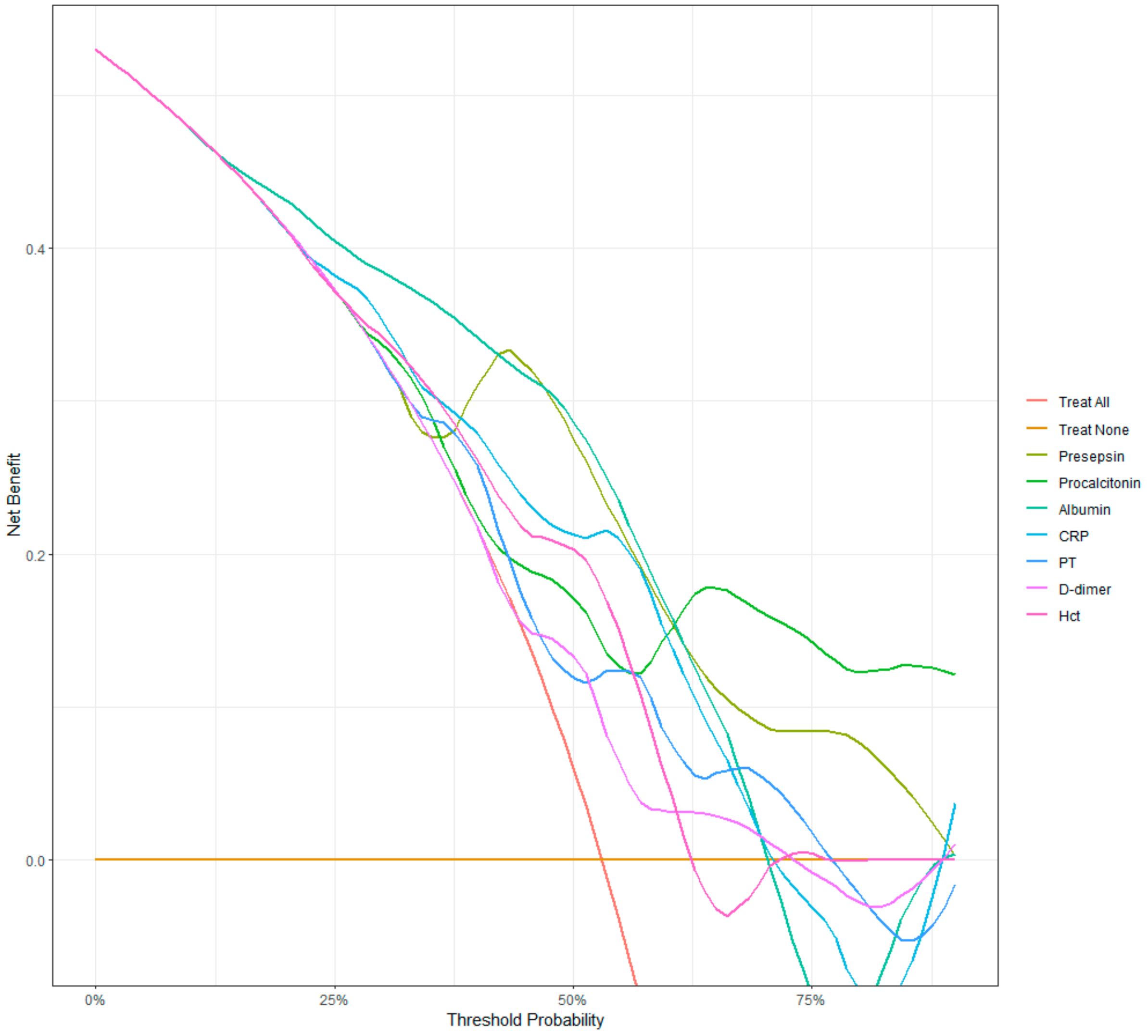

Figure S3. Decision curve analysis for diagnostic biomarkers in sepsis detection among burn patients with positive blood cultures.

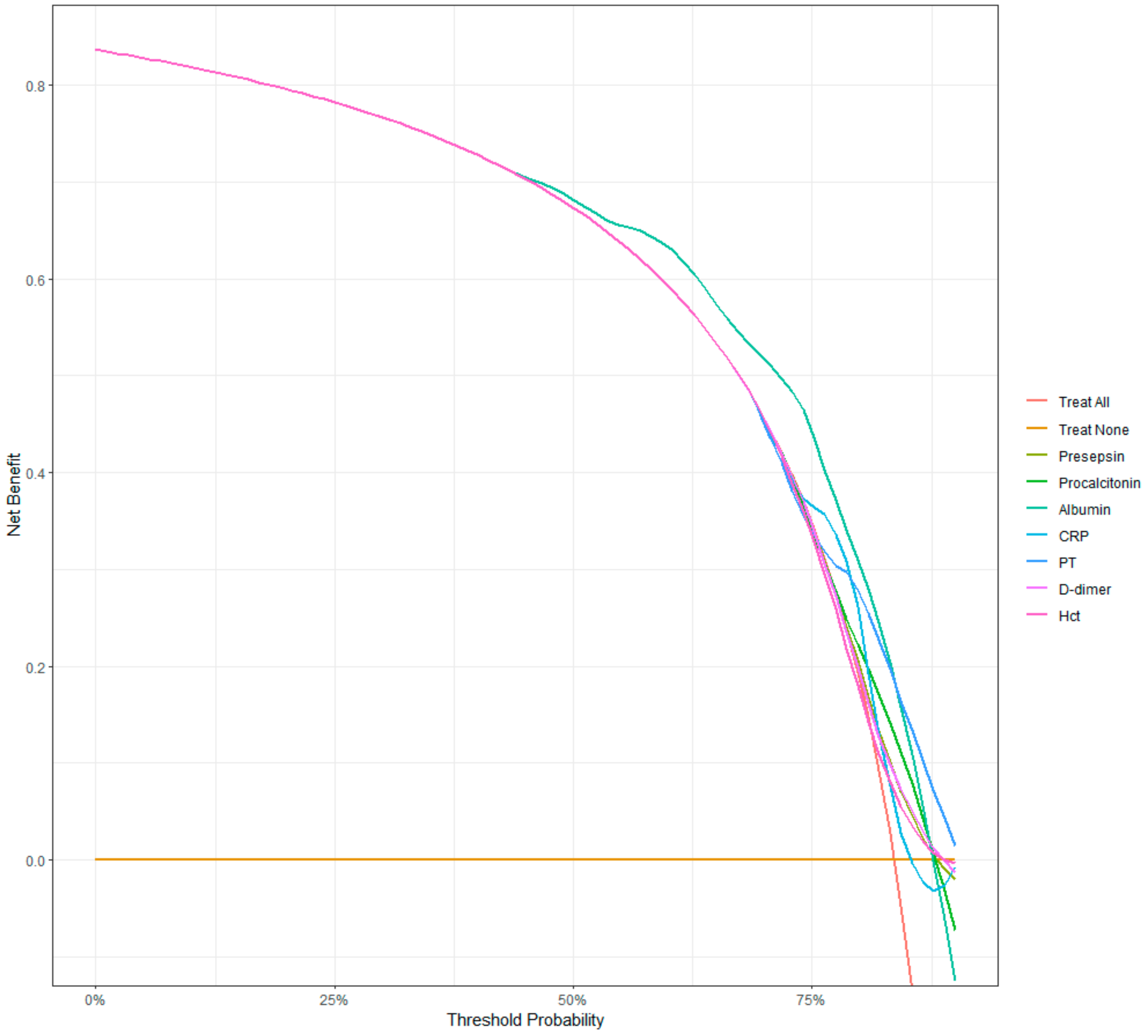

Figure S4. Kaplan–Meier survival curves comparing high and low biomarker groups over 60 days.

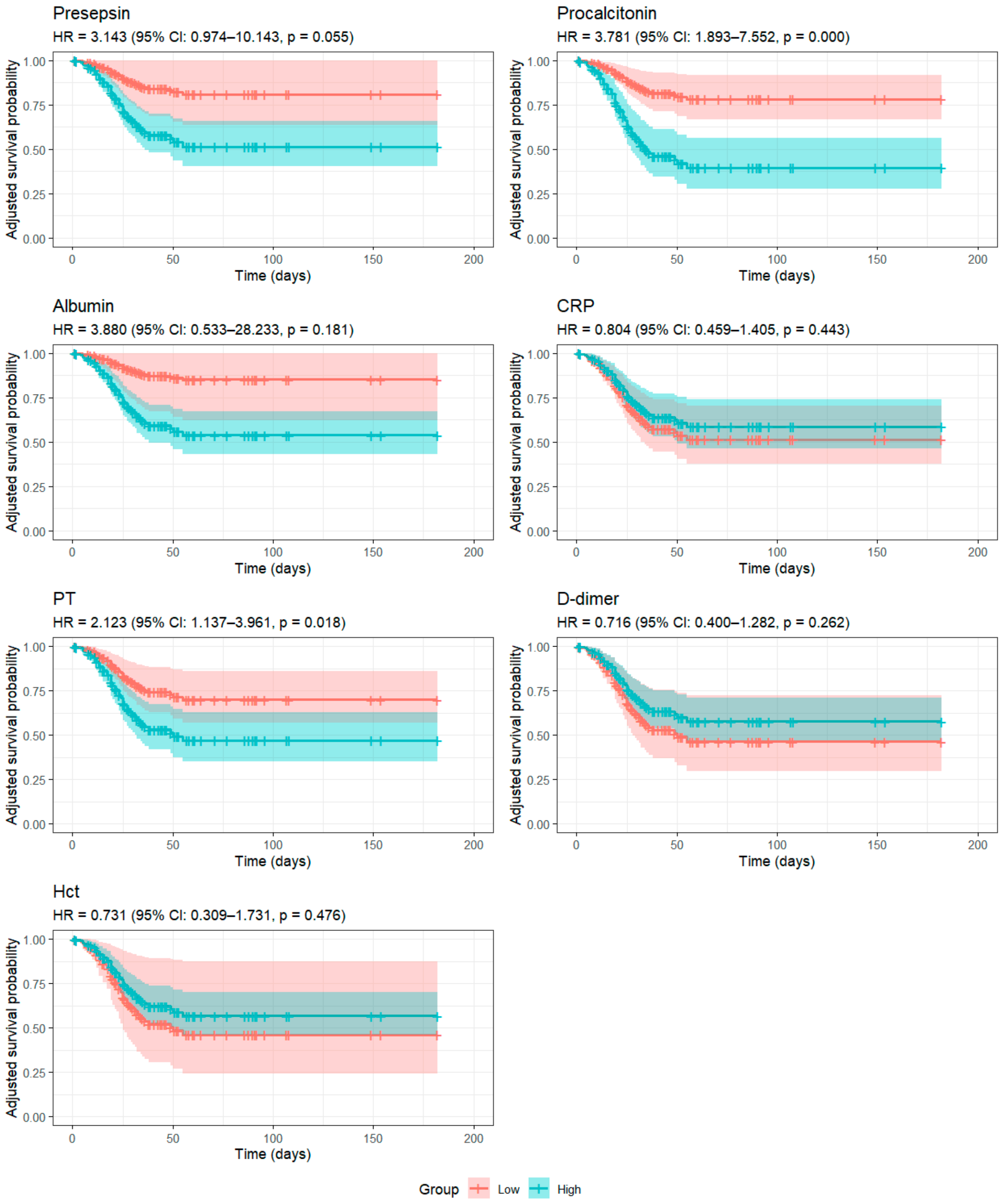

Table S1. Comparison of Random Forest and AUC analysis results for biomarker selection.

Rank	Variable	Importance	Rank	Variable	AUC
1	Presepsin	18.561	1	Presepsin	0.810
2	Albumin	13.546	2	Procalcitonin	0.752
3	Procalcitonin	10.177	3	Albumin	0.750
4	CRP	8.371	4	CRP	0.692
5	Hct	5.655	5	PT	0.685
6	Lactate	4.242	6	DB	0.680
7	PT	4.238	7	D-dimer	0.676
8	Creatinine	3.446	8	Cystatin	0.670
9	pH	3.270	9	Hct	0.669
10	Cystatin	3.236	10	pCO2	0.650
11	D-dimer	3.232	11	RDW	0.650
12	total Calcium	3.217	12	TB	0.650
13	pCO2	2.865	13	Lymphocyte	0.644
14	TB	2.804	14	Creatinine	0.642
15	RDW	2.610	15	aPTT	0.638
16	Glucose	2.584	16	Lactate	0.634
17	BUN	2.524	17	total Calcium	0.628
18	DB	2.070	18	pH	0.619
19	pO2	1.871	19	MPV	0.614
20	Lymphocyte	1.831	20	PCT	0.605
21	FDP	1.804	21	Platelet	0.605
22	aPTT	1.765	22	pO2	0.603
23	Antithrombin III	1.720	23	BUN	0.601
24	WBC	1.678	24	Potassium	0.596
25	AST	1.528	25	FDP	0.595
26	ALT	1.443	26	Fibrinogen	0.589

Rank	Variable	Importance	Rank	Variable	AUC
27	PCT	1.364	27	Monocyte	0.581
28	Potassium	1.346	28	PDW	0.579
29	CK	1.159	29	LD	0.578
30	Platelet	1.087	30	Sodium	0.573
31	MPV	0.802	31	AST	0.564
32	LD	0.564	32	serum Myoglobin	0.562
33	Neutrophil	0.495	33	CK	0.546
34	Monocyte	0.370	34	Phosphate	0.542
35	Fibrinogen	0.004	35	Bicarbonate	0.539
36	Phosphate	-0.058	36	Transferrin	0.522
37	Bicarbonate	-0.314	37	Antithrombin III	0.521
38	serum Myoglobin	-0.389	38	Glucose	0.508
39	Sodium	-0.711	39	ALT	0.507
40	Transferrin	-1.307	40	Neutrophil	0.498
41	PDW	-1.482	41	WBC	0.488

Rankings of biomarkers based on Random Forest importance scores and AUC values. Variables in red indicate common significant predictors identified by both methods. Shaded rows represent top 10 variables in each analysis. Random Forest importance score indicates variable's predictive power based on mean decrease in Gini impurity, while AUC values reflect discriminative ability of each variable.

Abbreviations: AUC, area under the curve; RF, Random Forest.

Table S2. ROC curves by culture status whose AUC differences between culture-negative and culture-positive groups reached statistical significance.

Table. Comparison of ROC Curves by Culture Status

		**ROC Analysis**		**DeLong's test**
**Biomarker**	**Culture Status**	**AUC (95% CI)**	**AUC Difference**	**P-value**
Presepsin	Positive	0.604 (0.425-0.783)	0.242	0.015 *
	Negative	0.846 (0.775-0.917)		
CRP	Positive	0.440 (0.273-0.608)	0.291	0.003 **
	Negative	0.732 (0.638-0.825)		

Significance codes: *** p < 0.001, ** p < 0.01, and * p < 0.05.

Table S3. Net benefit at different threshold probabilities in blood cultures overall.

	Threshold Probability														
**Biomarker**	**20%**	**25%**	**30%**	**35%**	**40%**	**45%**	**50%**	**55%**	**60%**	**65%**	**70%**	**75%**	**80%**	**85%**	**90%**
Presepsin	0.593	0.566	0.535	0.499	0.457	0.408	0.348	0.276	0.412	0.238	0.113	0.090	0.050	0.045	-0.027
Procalcitonin	0.593	0.566	0.535	0.499	0.457	0.408	0.348	0.276	0.299	0.244	0.170	0.113	0.100	0.071	0.009
Albumin	0.593	0.582	0.560	0.541	0.517	0.494	0.462	0.437	0.385	0.301	0.223	0.109	-0.018	-0.124	-0.036
CRP	0.593	0.566	0.535	0.499	0.457	0.437	0.389	0.358	0.278	0.229	0.157	0.068	-0.041	-0.066	0.000
PT	0.593	0.566	0.535	0.499	0.457	0.408	0.385	0.286	0.238	0.205	0.175	0.100	0.109	0.011	-0.014
D-dimer	0.593	0.566	0.535	0.499	0.457	0.408	0.348	0.276	0.186	0.178	0.066	0.054	0.032	-0.008	0.000
Hct	0.593	0.566	0.522	0.503	0.469	0.434	0.389	0.326	0.305	0.240	0.122	0.005	0.054	0.000	0.000

Table S4. Net benefit at different threshold probabilities in negative blood culture.

	Threshold Probability														
**Biomarker**	**20%**	**25%**	**30%**	**35%**	**40%**	**45%**	**50%**	**55%**	**60%**	**65%**	**70%**	**75%**	**80%**	**85%**	**90%**
Presepsin	0.412	0.373	0.328	0.277	0.348	0.333	0.282	0.213	0.158	0.114	0.088	0.077	0.077	0.046	0.000
Procalcitonin	0.412	0.373	0.328	0.296	0.222	0.174	0.162	0.114	0.154	0.172	0.160	0.145	0.120	0.137	0.120
Albumin	0.432	0.405	0.387	0.368	0.342	0.315	0.291	0.228	0.150	0.110	0.031	-0.077	-0.154	0.011	0.000
CRP	0.412	0.373	0.359	0.304	0.282	0.235	0.214	0.210	0.124	0.085	0.014	-0.051	-0.077	0.000	0.000
PT	0.412	0.373	0.328	0.277	0.279	0.155	0.094	0.162	0.077	0.063	0.046	0.017	-0.026	-0.054	0.000
D-dimer	0.412	0.373	0.328	0.277	0.217	0.145	0.162	0.047	0.034	0.028	0.011	-0.009	-0.026	0.000	0.000
Hct	0.412	0.365	0.335	0.308	0.265	0.205	0.214	0.138	0.043	-0.045	0.006	0.000	0.000	0.000	0.000

Table S5. Net benefit at different threshold probabilities in positive blood culture.

	Threshold Probability														
**Biomarker**	**20%**	**25%**	**30%**	**35%**	**40%**	**45%**	**50%**	**55%**	**60%**	**65%**	**70%**	**75%**	**80%**	**85%**	**90%**
Presepsin	0.796	0.782	0.766	0.749	0.728	0.703	0.673	0.637	0.591	0.533	0.455	0.346	0.183	0.093	0.000
Procalcitonin	0.796	0.782	0.766	0.749	0.728	0.703	0.673	0.637	0.591	0.533	0.455	0.346	0.183	0.122	-0.067
Albumin	0.796	0.782	0.766	0.749	0.728	0.703	0.683	0.649	0.635	0.577	0.503	0.442	0.288	0.106	-0.106
CRP	0.796	0.782	0.766	0.749	0.728	0.703	0.673	0.637	0.591	0.533	0.455	0.346	0.327	-0.035	0.000
PT	0.796	0.782	0.766	0.749	0.728	0.703	0.673	0.637	0.591	0.533	0.455	0.317	0.317	0.083	-0.000
D-dimer	0.796	0.782	0.766	0.749	0.728	0.703	0.673	0.637	0.591	0.533	0.455	0.346	0.183	0.054	-0.019
Hct	0.796	0.782	0.766	0.749	0.728	0.703	0.673	0.637	0.591	0.533	0.455	0.327	0.163	0.151	0.000

Table S6. Comparison of IDI and NRI with presepsin in negative blood culture.

Biomarker	IDI (95% CI)	P-value	NRI (95% CI)	P-value
Procalcitonin	0.009 (-0.375 ~ 0.392)	0.821	0.067 (-0.773 ~ 0.906)	0.716
Albumin	0.059 (-0.337 ~ 0.455)	0.146	0.434 (-0.398 ~ 1.266)	**0.016 ***
CRP	-0.030 (-0.413 ~ 0.352)	0.424	0.010 (-0.833 ~ 0.853)	0.957
PT	-0.096 (-0.455 ~ 0.262)	**0.004 ****	-0.374 (-1.206 ~ 0.459)	**0.038 ***
D-dimer	-0.136 (-0.502 ~ 0.231)	**<0.001 *****	-0.838 (-1.617 ~ -0.060)	**<0.001 *****
Hct	-0.074 (-0.440 ~ 0.291)	**0.033 ***	-0.172 (-1.009 ~ 0.665)	0.346

Significance codes: *** p < 0.001, ** p < 0.01, and * p < 0.05.

Table S7. Comparison of IDI and NRI with presepsin in positive blood culture.

Biomarker	IDI (95% CI)	P-value	NRI (95% CI)	P-value
Procalcitonin	0.007 (-0.175 ~ 0.189)	0.414	0.150 (-0.787 ~ 1.087)	0.511
Albumin	0.068 (-0.282 ~ 0.418)	**0.033** *	0.441 (-0.559 ~ 1.441)	0.090
CRP	0.007 (-0.212 ~ 0.226)	0.582	-0.070 (-1.079 ~ 0.938)	0.791
PT	0.027 (-0.213 ~ 0.267)	0.071	0.679 (-0.264 ~ 1.622)	**0.003** **
D-dimer	0.005 (-0.191 ~ 0.202)	0.599	0.216 (-0.753 ~ 1.186)	0.377
Hct	-0.001 (-0.160 ~ 0.158)	0.915	0.183 (-0.824 ~ 1.189)	0.488

Significance codes: *** $p < 0.001$, ** $p < 0.01$, and * $p < 0.05$.

Table S8. Clinical characteristics of sepsis patients: implications for mortality prediction.

Group	Variables	**Overall**, N = 149	**Non-survival**, N = 59 (39.6%)	**Survival**, N = 90 (60.4%)	**p-value**
Outcomes	Positive blood culture	87 (58.4%)	42 (71.2%)	45 (50.0%)	0.011
Base	Age				0.015
	Median [IQR]	56 [44, 66]	61 [51, 69]	55 [38, 62]	
	Sex				0.265
	Male	125 (83.9%)	47 (79.7%)	78 (86.7%)	
	Female	24 (16.1%)	12 (20.3%)	12 (13.3%)	
	TBSA (%)				<0.001
	Median [IQR]	42 [25, 60]	57 [35, 76]	40 [22, 48]	
	Inhalation				0.214
	No	119 (79.9%)	44 (74.6%)	75 (83.3%)	
	Yes	30 (20.1%)	15 (25.4%)	15 (16.7%)	
	Type				0.344
	FB	117 (78.5%)	47 (79.7%)	70 (77.8%)	
	SB	14 (9.4%)	8 (13.6%)	6 (6.7%)	
	EB	10 (6.7%)	2 (3.4%)	8 (8.9%)	
	ChB	1 (0.7%)	0 (0.0%)	1 (1.1%)	
	CoB	7 (4.7%)	2 (3.4%)	5 (5.6%)	
	LOICU (days)				<0.001
	Median [IQR]	25 [18, 38]	19 [13, 27]	30 [20, 52]	
Medical history	Cardiovascular disease	29 (19.5%)	15 (25.4%)	14 (15.6%)	0.145
	Endocrine disorders	15 (10.1%)	5 (8.5%)	10 (11.1%)	0.782

Group	Variables	**Overall**, N = 149	**Non-survival**, N = 59 (39.6%)	**Survival**, N = 90 (60.4%)	**p-value**
	Malignant	8 (5.4%)	7 (11.9%)	1 (1.1%)	0.007
	Others	2 (1.3%)	1 (1.7%)	1 (1.1%)	>0.999
	Operations	51 (34.2%)	22 (37.3%)	29 (32.2%)	0.597
Severity scores	ABSI				<0.001
	Median [IQR]	9 [8, 11]	11 [10, 13]	8 [7, 9]	
	rBaux				<0.001
	Median [IQR]	101 [91, 115]	115 [106, 133]	95 [82, 103]	
	Hangang				<0.001
	Median [IQR]	134 [123, 148]	150 [141, 168]	126 [117, 134]	
	APACHE IV				<0.001
	Median [IQR]	70 [47, 87]	83 [68, 105]	56 [37, 76]	
	SOFA				<0.001
	Median [IQR]	5 [3, 9]	10 [7, 12]	4 [3, 5]	
Complications	ARDS	56 (37.6%)	35 (59.3%)	21 (23.3%)	<0.001
	AKI	54 (36.2%)	43 (72.9%)	11 (12.2%)	<0.001
Interventions	Ventilator	101 (67.8%)	56 (94.9%)	45 (50.0%)	<0.001
	CRRT	44 (29.5%)	40 (67.8%)	4 (4.4%)	<0.001
Biomarkers	Presepsin				<0.001
	Median [IQR]	1,093 [559, 2,362]	2,239 [1,184, 7,782]	773 [469, 1,226]	
	Procalcitonin				<0.001
	Median [IQR]	0.7 [0.3, 2.2]	2.2 [0.9, 6.5]	0.4 [0.2, 0.8]	
	Albumin				<0.001
	Median [IQR]	2.50 [2.30, 2.70]	2.30 [2.10, 2.45]	2.50 [2.40, 2.70]	

Group	Variables	**Overall**, N = 149	**Non-survival**, N = 59 (39.6%)	**Survival**, N = 90 (60.4%)	**p-value**
	CRP				0.451
	Median [IQR]	130 [83, 190]	139 [82, 197]	125 [83, 177]	
	PT				<0.001
	Median [IQR]	15.10 [14.00, 16.90]	16.30 [15.10, 18.20]	14.45 [13.63, 15.30]	
	Cystatin				0.002
	Median [IQR]	1.00 [0.80, 1.50]	1.30 [0.90, 1.70]	0.90 [0.73, 1.20]	
	Hct				0.014
	Median [IQR]	27.7 [25.0, 30.5]	27.1 [23.6, 29.5]	28.1 [25.8, 31.0]	
